# Dietary Supplementation of Microalgae and/or Nanominerals Mitigate the Negative Effects of Heat Stress in Growing Rabbits

**DOI:** 10.1007/s12011-023-03953-0

**Published:** 2023-11-15

**Authors:** Amr M. Bashar, Sameh A. Abdelnour, Abdelhalim A. El-Darawany, Asmaa M. Sheiha

**Affiliations:** Department of Animal Production, Faculty of Agriculture, Zagazig University, Zagazig, 44511 Egypt

**Keywords:** *Spirulina platensis*, Nanominerals, Growth, Blood hematology, Serum metabolites, Antioxidant-immune responses

## Abstract

Heat stress (HS) is one of the most significant environmental factors that result in fluctuations and shrinkage in rabbit growth, health, and overall productivity. This study aims to investigate the effects of dietary mineral nanoparticles (selenium or zinc) and/or *Spirulina platensis* (SP) independently and in combination on stressed growing rabbits. A total of 180 weaned growing New Zealand White rabbits were included in this study and randomly divided into six dietary treatments. Rabbits received a basal diet (control group; CON group) or fortified with SP (1 g/kg diet), selenium nanoparticles (SeNPs, 50 mg/kg diet), zinc nanoparticles (ZnNPs, 100 mg/kg diet), and a mixture of SP and SeNPs (SPSeNPs) or SP and ZnNPs (SPZnNPs) groups for 8 weeks during summer conditions. The obtained results demonstrated a significant increase in the final body weight and weight gain (*p* < 0.05). Additionally, the feed conversion ratio was improved during the periods from 6 to 14 weeks in the treated rabbits compared to those in the CON group. Dietary supplements considerably improved (*p* < 0.05) the blood hematology (WBCs, Hb, RBCs, and Hct) and some carcass traits (liver weights and edible giblets). All dietary supplements significantly decreased serum levels of total glycerides (*p* < 0.0001), AST (*p* = 0.0113), ALT (*p* = 0.0013), creatinine (*p* = 0.0009), and uric acid (*p* = 0.0035) compared to the CON group. All treated groups (except ZnNPs) had lower values of total bilirubin and indirect bilirubin in a dose-dependent way when compared to the CON group. The values of IgA, IgG, and superoxide dismutase were significantly improved (*p* < 0.05) in all treated rabbits compared to the CON group. Compared with the CON group, the levels of T3 (*p* < 0.05) were significantly increased in all treated growing rabbits (except for the ZnNP group), while the serum cortisol, interferon-gamma (IFN-γ), malondialdehyde, and protein carbonyl were significantly decreased in the treated groups (*p* < 0.05). Dietary supplements sustained the changes in hepatic, renal, and cardiac impairments induced by HS in growing rabbits. Adding SP (1 g/kg diet) or SeNPs (50 mg/kg diet) in the diet, either individually or in combination, improved growth performance, blood picture, and immunity-antioxidant responses in stressed rabbits. Overall, these feed additives (SP, SeNPs, or their mixture) can be applied as an effective nutritional tool to reduce negative impacts of summer stress conditions, thereby maintaining the health status and improving the heat tolerance in growing rabbits.

## Introduction

Global warming poses a major threat to the livestock industry’s sustainability and profitability [1]. This phenomenon is related to the rise in ambient temperatures, which leads to heat stress (HS) [2]. In this sense, HS can lead to reduced feed efficiency, decreased growth, lower milk yield, impaired reproduction, and increased susceptibility to diseases of various livestock species [3, 4]. HS can diminish the health and immunity of rabbits resulting in increasing the mortality rates in rabbit farms [5, 6]. In the last decades, the rabbit industry could contribute in an effective way to meat production as their meat is considered a good source of many elements such as protein, calories, and minerals and vitamins. The post-weaning stage is a critical period in the productive cycle of the rabbit industry. Growing rabbits can be raised for meat production and are often used for culling in rabbit farms. In addition to weaning stress affecting the growing rabbits, the HS could also impact on the health and welfare [7].

It is a relatively small-scale industry, but it is growing in popularity in some parts of the world. Rabbits are highly sensitive to high temperatures [8] due to their inability to effectively regulate their body temperature during HS [7]. Prolonged exposure to high temperatures can lead to hyperthermia and an increase in body temperature [9]. Rabbits are more prone to heat stroke compared to many other animals [7], because they have a limited ability to sweat and cool themselves down [8]. To reduce the negative effects of HS on rabbits, many nutritional strategies have been implemented for this purpose [10–12].

Selenium is an essential trace element for rabbits. It plays significant roles in many important body functions such as reproduction, immunity, and antioxidant defense [13]. Recently, selenium nanoparticles (SeNPs) have gained attention due to their numerous advantages, including low cytotoxicity, increased surface area for interacting with biological targets, and high drug-loading capacity [5, 14].

Consequently, SeNPs appear to be of great importance in rabbit nutrition. Moreover, SeNPs can help maintain cellular functions by protecting against lipid peroxidation, and oxidative stress, enhance immunity, and indirectly promote growth performance in broilers [13, 15, 16].

Zinc (Zn) is another one of the essential trace minerals. It has been widely incorporated into animal diets as growth promoters. Its inclusion has the potential to improve growth, bone development, immune system, and organ functions [17, 18]. Zn plays a crucial role in the metabolism of protein, fat, and carbohydrates, as well as in maintaining gut health in poultry [19, 20]. Additionally, it contributes to improved feed efficiency [19]. By adding zinc oxide (100 mg/kg) to the diets, the performance, carcass yield, and antioxidant response of growing rabbits can be improved under high-temperature conditions [12]. However, the high phytate content in rabbit feed ingredients may reduce Zn absorption [21]. Introducing a new type of Zn could tackle this problem. Zinc nanoparticles (ZnNPs) are a new type of nanomaterial that has shown potential benefits for rabbits during HS [11, 22]. ZnNPs have robust antioxidant activity, making them beneficial effects for reducing the negative effects of hot weather in broilers [23, 24] and rabbits [22]. *Spirulina platensis* (SP), a filamentous cyanobacterium, is a nutrient-rich microalgae with a wide range of therapeutic properties, health benefits, and biological activities [25]. These properties may be attributed to a variety of bioactive compounds including phycocyanin, carotenoids, polysaccharides, and phenolic, which are present in SP [26]. The biological activities of SP including immunostimulatory, hepatoprotective, antioxidative, and antiinflammatory actions have been clarified [26]. This makes SP a potential candidate for reducing HS in rabbits via decreasing inflammation/oxidative stress [10] and for preventing and treating a variety of diseases [25]. SP has also been shown to improve health and immunity as well as reduce inflammation/oxidative stress induced by HS in broilers and rabbits [12, 15, 16].

Nanotechnology and its related products have made rapid progress in recent years in various scientific fields. Nanominerals have been shown to interact more efficiently than organic and inorganic materials in the animal body. This is due to their larger surface area, solid adsorption ability, and high catalytic competence [27]. As a result, nanoscale minerals are expected to be more efficient, bioavailable, and interactive compared to traditional mineral sources. A few studies have demonstrated the beneficial effects of adding ZnNPs [11, 22] or SeNPs [5] on the health and welfare of stressed growing rabbits. Previous research by [5, 28], and [12], used doses of SP (1 g/kg diet), SeNPs (50 mg/kg diet), and SeNPs (100 mg/kg diet) in rabbit diets to improve growth and health. However, there is a lack of comparative studies on these nanominerals when incorporated with SP in stressed rabbits. Thereafter, this study aimed to examine the effects of dietary nanomineral [zinc nanoparticles (ZnNPs) or selenium nanoparticles (SeNPs)] when incorporated with SP on the growth, feed utilization, blood hematology, serum biochemistry, antioxidant-immune response, and carcass yield of growing rabbits reared under summer conditions.

## Material and Methods

### Ethical Approval

The current trial was carried out at the Animal Production Department, Faculty of Agriculture, Zagazig University, Egypt. This experiment was carried out in agreement with animal ethics and approved by the Zagazig University Institutional Animal Care and Use Committee (Approval No. ZUIACUC/2//F/61/2016).

### Material Sources and Characterization

The dried *Spirulina platensis* (SP) was acquired from the SCAD Company for agricultural development, while the sodium selenite and zinc oxide were acquired from Algomhoria Company, Zagazig, Egypt.

#### Selenium Nanoparticle (SeNP) Synthesis

A pure lyophilized culture of the *Lactobacillus plantarum* strain was acquired from the Department of Microbiology, Faculty of Agriculture, Zagazig University. The bacterial culture (2%) with approximately 10^5^ cfu/mL was cultivated in an MRS broth medium. The medium was then enriched with 0.1% skim milk powder and 1% yeast extract. Sodium selenite (Algomhoria Company) was added to the media, which was incubated aerobically at 37 °C for 3–4 days in Erlenmeyer flasks without agitation. The mixture was used to detect the size of SeNPs, which were then exposed to lyophilization, weighed, and added to the diets [16] according to the study protocol.

#### Zinc Oxide Nanoparticle (ZnNP) Synthesis

**Fig. 1:**
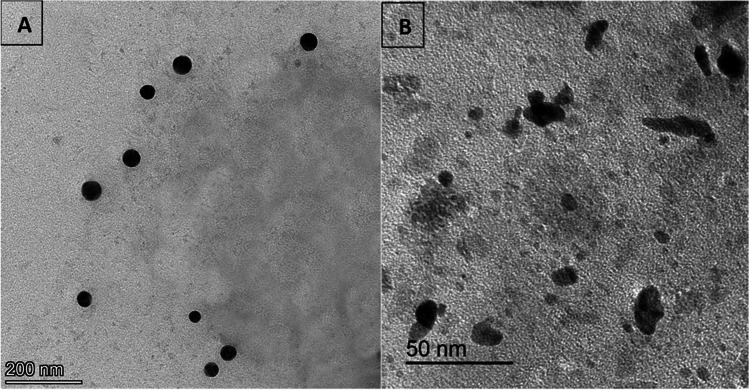
Transmission electron microscopy (TEM) images of selenium nanoparticles (**A**) and zinc oxide nanoparticles (**B**). The selenium nanoparticles have an oval and spherical shape, whereas the zinc oxide nanoparticles have an irregular shape

Zinc oxide was obtained from the Algomhoria Company, Zagazig, Egypt. The fresh bacterial strain *L. plantarum* was maintained on a nutrient agar medium at 37 °C for 24 h in a 250-mL Erlenmeyer flask. The suspension was enriched with sterilized deionized water containing zinc oxide. The culture was subcultured into a nutrient broth medium and constantly shaken at 150 rpm at 37 °C for 24 h. The supernatant was collected after centrifugation at 5000 rpm for 5 min in an overnight bacterial culture and it was used for the synthesis of ZnNPs. The collected ZnNPs were dried at 100 °C and used in this study based on a previous method [11]. Fig. 1 shows the TEM of selenium nanoparticles (Fig. 1A) and zinc oxide nanoparticles (Fig. 1B).

**Fig. 2:**
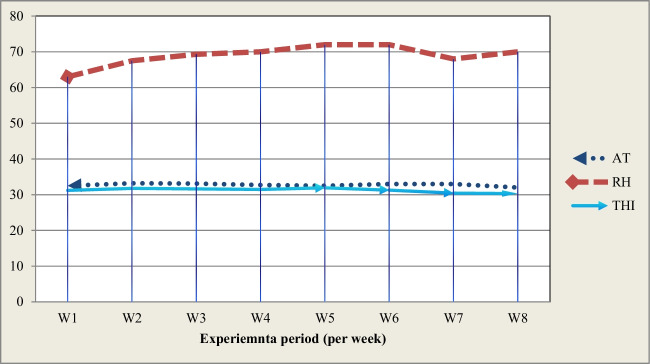
The values of THI, RH, and AT during the 8 weeks of the experimental period at the study location (Zagazig, Egypt)

### Meteorological Measurements

This study lasted for 8 weeks during the natural summer conditions at Zagazig City, Al-Sharkia Governance, Egypt. In order to assess the severity of HS on growing rabbits, we used the THI (temperature humidity index) equation;

$$\textrm{THI}=\textrm{db}-\left[\right(0.31-0.31\left(\textrm{RH}\right)\Big]\times \left[\left(\textrm{db}{}^{\circ}\textrm{C}-14.4\right)\right],$$

where RH is the relative humidity and db is the dry bulb temperature in Celsius.

The measured THI values were accordingly classified as follows:

- <27.8 (absence of HS);
- 27.8 to 28.9 (moderate HS);
- 28.9 to 30.0 (severe HS);
- and >30.0 (extremely severe HS) [8]

### Rabbits and Experimental Protocol

A total of 180 weaned New Zealand White male rabbits aged approximately 35 days with an average body weight of 716.9±12g were enrolled in the existing study at the farm of the Animal Production Department, Faculty of Agriculture, Zagazig University, Egypt. Rabbits were randomly divided into 6 equal experimental groups, with each group consisting of 30 rabbits and 15 replicates. The rabbits were either given a basal diet (CON group) or fed experimental diets fortified with *Spirulina platensis* (SP, 1gkg diet) [28], selenium nanoparticles (SeNPs, 50 mg/kg diet) [5], zinc nanoparticle (ZnNPs, 100 mg/kg diet) [12], or a mixture between SP + SeNPs or SP + ZnNPs, respectively. The feed additives were added to the diets during the feed manufacturing process, following the study protocol. All the growing rabbits were housed in standard cages and fed pelleted diets. They had free access to water and feed throughout the study period. The diets were formulated based on the nutrient recommendations for rabbits supported by [29]. The diet constituents and the chemical composition of the basal experimental diet are listed in Table 1.

**Table 1:** The ingredients and chemical composition of the basal diet

Items	Basal diet
Ingredient	%
Soybean meal 44%	20
Barley grain	10
Maize	20
Wheat bran	16
Berseem hay	30
Molasses	2
Limestone	1
NaCl and premix*	1 (0.5 and 0.5)
Chemical analysis (%, on DM basis) ^**^	
Dry matter	88.36
Crude fiber	14.2
Crude protein	17.21
Ether extract	2.32
Ash	9.26
Organic matter	90.74

*Each 1 kg of premix (minerals and vitamins mixture) contains vit. D_3_, 15,000 IU; vit. A, 20,000 IU; vit. E, 8.33 g; vit. B_1_, 0.33 g; vit. K, 0.33 g; vit. B_2_, 1.0 g; vit. B_5_, 8.33 g; vit. B_6_, 0.33 g; vit. B_12_, 1.7 mg; folic acid, 0.83 g; biotin, 33 mg; choline chloride, 200 g; pantothenic acid, 3.33 g; Cu 0.1 mg, Fe 75.0 mg, Mn 8.5 mg, ZnO 20 mg, iodine 0.2mg, Co 0.5mg, Mg 8.5 mg 0.1 mg sodium selenite. **Chemical analysis according to [30]

### Growth Performance

The rabbits were individually weighed using digital balance, and the feed intakes (FI) were also recorded during the experimental period. The live body weights (BW) and daily body weight gain (BWG) were estimated at two periods (at 10 and 14 weeks of age), but only averages of BW at 6 (start point), 10 (4 weeks of treatments), and 14 (end point) weeks of ages were documented. The feed intakes (FI) and feed conversion ratio (FCR, g feed/g BWG) for the entire experimental period were calculated at the periods 6–10 weeks, 10–14 weeks, and the overall 6–14 weeks of age.

### Sampling and Blood Hematological Variables

At the end of the experiment, ten rabbits per group were randomly selected to pick up the blood samples from the ear vein following the method described in [31]. The blood samples were divided into two subsamples. The first one was collected in a clean centrifuge tube without anticoagulant for serum separation of serum. It was then centrifuged at 2000 gravity for 20 min and stored at −20 °C for biochemical analyses. The second subsample was collected in EDTA-containing tubes and used to assess hematological variables in the whole blood. The collected whole blood was used for determining the hemoglobin (Hb), red blood cells (RBCs), hematocrit (Hct), mean corpuscular hemoglobin concentration (MCHC), mean cell volume (MCV), mean corpuscular hemoglobin (MCH), platelets, mean platelet volume (MPV), platelet distribution width (PDW), and white blood cells (WBCs) using DxH 900 Hematology Analyzer (C23653 - DxH 900-2, Beckman Coulter, USA).

### Blood Metabolites

Serum metabolites of rabbits including total protein (TP), albumin (ALB), glucose, total triacylglycerol (TG), alanine aminotransferase (ALT), creatinine, uric acid, aspartate aminotransferase (AST), total bilirubin (TB), direct bilirubin (DB), and indirect bilirubin (IDB) were determined spectrophotometrically using a commercial diagnostic kit (Spinreact, Santa Coloma, Spain). The globulin (GLU) was calculated by subtracting albumins from total proteins, while A/G was calculated by dividing the ALB/GLU.

### Antioxidant-Immune Responses

ELISA kits provided by Elabscince Company (Houston, TX, 77079, USA) were used to assess the levels of immunoglobulins (IgA and IgM) in the serum of rabbits [32] following the directions protocols. The serum levels of malondialdehyde (MDA; ab27642) were assessed based on the reaction with thiobarbituric acid [33] and protein carbonyl (PC; ab126287) were assessed using commercial ELISA kits (Abcam company) based on the reaction with DNPH [34]. Glutathione (GSH) and superoxide dismutase (SOD) were assessed using commercial kits provided by Nanjing Jiancheng Bioengineering Institute (Nanjing, China), following the recommendations in the pamphlet. The serum level of interferon-gamma (IFN-γ; ab273238) was assessed using a colorimetric technique with a commercial diagnostic kit (Abcam company), which had a sensitivity of 0.16 ng/mL [35]. The levels of serum triiodothyronine (T3) and corticosterone were measured using commercially available assays. T3 levels were determined using a direct ELISA assay from Diametra Srl, Segrate, Italy. Corticosterone levels were determined using corticosterone ELISA kits [36], following the manufacturer’s recommendations (Assay Pro-LLC, Saint Charles, MO, USA).

### Carcass Traits

At the end of the experiment period, ten rabbits from each group were randomly selected. They were fasted overnight, weighed, and euthanized by cervical dislocation, in accordance with the AVMA Guidelines [37]. The dressing, liver, heart, kidney, lung, spleen, edible giblets, tests, total edible parts, and non-edible parts were also expressed as a percentage of the slaughter weight. The weight of certain organs was considered in accordance with [4].

### Histological Study

Animals (*n* = 5) from each treatment were sacrificed to study the histological alterations in hepatic, renal, and cardiac tissues after ending the experiment. Specimens were obtained, cleaned with distilled water, and then carefully fixed in a 10% neutral buffered formalin solution for 3 days. After fixation, administered using the paraffin procedure, partitioned at 4–5 μm, stained with hematoxylin-eosin, and examined microscopically [38]. The stained slides were scrutinized microscopically for morphological modifications.

### Statistical Analyses

The results were statistically analyzed using SPSS® software version 21 (SPSS, Chicago, IL, USA), following the one-way ANOVA analysis and the post hoc Newman–Keuls test. Differences were considered statistically significant at *p* < 0.05. The statistical model applied in this study is: *Y*_ijk_ = *μ* + *T*_i_ + *e*_ijk_ where *Y* is either the trait; *T*_i_ is the effect of treatment, *μ* is the mean of the trait, and *e*_ijk_ is the error respectively.

## Results

### Meteorological Measurements

As clarified in Fig. 2, the values of THI were 31.25 all over the period, indicating the severity of HS on growing rabbits. Moreover, during the first 6 weeks, it appears that the THI values were higher compared to the last 2 weeks of the experiment period.

### Effects on Growth Indices

At 10 weeks, the BW was significantly improved in all supplemented groups (except the ZnNPs group) (*p* < 0.0001). The CON and ZnNP groups showed similar results (*p* > 0.05; Table 2). By 14 weeks of treatment, the SPSeNP group exhibited the best results for BW. However, the ZnNP group had the lowest BW among all the treated groups with no significant differences recorded among the SP, SeNP, and SPZnNP groups. All treated groups had a significant effect on BWG (*p* < 0.0001) during the 6–10 weeks period compared to the CON group. The ZnNP group exhibited intermediate values for DWG. At 10–14 weeks, SPZnNPs showed the best results for DWG without a significant difference compared to the SeNP and ZnNP groups. Regarding the overall period (6–14 weeks), BWG was greater in the SPSeNP group (*p* < 0.01) compared to the other groups. Additionally, all treated groups significantly improved DWG over the 6–14-week period (*p* = 0.0051).

**Table 2:** Effects of nanominerals (SeNPs or ZnNPs) either alone or incorporated with *Spirulina platensis on* (SP) on body weight (g) and daily weight gain (g) of growing rabbits under high elevated conditions

Treatments^1^	Body weight (BW, g)			Daily weight gain (DWG, g/day)		
	6 weeks	10 weeks	14 weeks	6–10 weeks	10–14 weeks	6–14 weeks
CON	719.80	1473.30^**b**^	2046.20^**c**^	26.91^**c**^	20.46^**b**^	23.69^**c**^
SP	712.70	1563.80^**a**^	2187.70^**ab**^	30.40^**a**^	22.28^**b**^	26.34^**ab**^
SeNPs	719.30	1549.10^**a**^	2197.69^**ab**^	29.64^**a**^	23.16^**ab**^	26.40^**ab**^
SPSeNPs	720.50	1564.10^**a**^	2240.50^**a**^	30.13^**a**^	24.16^**a**^	27.14^**a**^
ZnNPs	716.40	1504.20^**b**^	2152.90^**b**^	28.14^**b**^	23.17^**ab**^	25.65^**b**^
SPZnNPs	712.70	1566.90^**a**^	2190.70^**ab**^	30.51^**a**^	22.28^**b**^	26.39^**ab**^
SEM	5.68	11.71	17.76	0.34	0.48	0.30
*p*-value	0.8548	<0.0001	<0.0001	0.0186	0.0001	0.0051

^1^Rabbit given basal diet (CON group) or supplemented with *Spirulina platensis* (SP; 1g/kg diet), selenium nanoparticles (SeNPs, 50 mg/kg diet), zinc nanoparticles (ZnNPs; 100 mg/kg diet), SPSeNPs (SeNPs + SP), and SPZnNPs (ZnNPs + SP). Means within a column not sharing a common superscript differ (*p* < 0.05); SEM standard error of means

### Effects on Feed Utilization

As reported in Table 3, feed intake did not differ among all treatments at all periods (*p* > 0.05. Compared with the SP group, feed intake was greater in the CON group (*p* < 0.05). While the SP group had inferior feed intake compared with the other treated groups (*p* > 0.05) at the period 6–10 weeks. The dietary inclusion of SeNPs, SPSeNPs, or ZnNPs significantly improved the FCR (*p* < 0.001). Specifically, the addition of SeNPs improved the FCR during the overall 6–14 week period (*p* = 0.0293). FCR did not differ (*P >* 0.05) among the CON and other treated groups

**Table 3:** Effects of nanominerals (SeNPs or ZnNPs) either alone or incorporated with *Spirulina platensis* (SP) on total feed intake (g/day) and feed conversion ratio (g feed/g gain) of growing rabbits under summer conditions

Treatments^1^	Feed intake (g/day)			Feed conversion ratio (g feed/g gain)		
	6–10 weeks	10–14 weeks	6–14 weeks	6–10 weeks	10–14 weeks	6–14 weeks
CON	77.71	100.50	91.80	2.89^**a**^	4.91^**a**^	3.88^**a**^
SP	81.80	107.00	94.70	2.69^**b**^	4.81^**ab**^	3.59^**ab**^
SeNPs	82.60	105.80	93.20	2.79^**ab**^	4.57^**bc**^	3.53^**b**^
SPSeNPs	82.30	109.00	98.70	2.73^**ab**^	4.51^**bc**^	3.64^**ab**^
ZnNPs	81.30	100.20	94.60	2.90^**ab**^	4.32^**c**^	3.71^**ab**^
SPZnNPs	82.30	108.00	95.70	2.70^**ab**^	4.85^**ab**^	3.63^**ab**^
SEM	1.85	4.97	2.82	0.07	0.11	0.12
*p*-value	0.6897	0.0661	0.7913	0.0464	0.0003	0.0293

^1^Rabbit were given basal diet (CON group) or supplemented with *Spirulina platensis* (SP; 1g/kg diet), selenium nanoparticles (SeNPs, 50 mg/kg diet), zinc nanoparticles (ZnNPs; 100 mg/kg diet), SPSeNPs (SeNPs + SP), and SPZnNPs (ZnNPs+SP). Means within a column not sharing a common superscript differ (*p* < 0.05); SEM standard error of means

### Effects on Carcass Traits

As presented in Table 4, the dietary treatments had no significant influence on all carcass traits (except for edible giblets and liver). All treated groups showed a significant improvement in liver weight compared to the CON group (*P* < 0.05). The same trend was observed for edible giblets.

**Table 4:** Effects of nanominerals (SeNPs or ZnNPs) either alone or incorporated with *Spirulina platensis* (SP) on carcass traits of growing rabbits under summer conditions

Item	Treatments^3^						SEM	*p*-value
	CON	SP	SeNPs	SPSeNPs	ZnNPs	SPZnNPs		
Pre-slaughter weight (g)	2092.00	2077.33	2066.33	2054.67	2084.00	2095.33	15.836	0.4216
Dressing (%)	59.98	60.93	61.36	61.72	60.05	59.85	1.49	0.9136
Liver (%)	2.79^**b**^	3.12^**a**^	3.37^**a**^	3.45^**a**^	3.41^**a**^	3.25^**a**^	0.14	0.0428
Heart (%)	0.29	0.30	0.32	0.29	0.27	0.35	0.05	0.8680
Kidney (%)	0.91	0.89	0.83	0.92	0.93	0.91	0.06	0.8976
Edible giblets (%)^1^	3.98^**b**^	4.31^**a**^	4.52^**a**^	4.66^**a**^	4.61^**a**^	4.51^**a**^	0.17	0.0231
Lung (%)	0.72	0.67	0.74	0.68	0.67	0.70	0.06	0.9571
Spleen (%)	0.05	0.08	0.12	0.11	0.10	0.13	0.02	0.3004
Tests (%)	0.20	0.27	0.27	0.28	0.26	0.22	0.01	0.2122
Total edible parts (%)^2^	63.97	65.24	65.88	66.38	64.65	64.36	1.49	0.8499
Non-edible parts (%)	36.03	34.76	34.12	33.62	35.35	35.64	1.49	0.8499

^1^Edible giblets [%] = (liver + kidney + heart)/pre-slaughter weight (g) × 100. ^2^Total edible parts [%] = (carcass weight + edible giblets weight)/pre-slaughter weight (g) × 100. ^3^Rabbit given basal diet (CON group) or supplemented with *Spirulina platensis* (SP; 1g/kg diet), selenium nanoparticles (SeNPs, 50 mg/kg diet), zinc nanoparticles (ZnNPs; 100 mg/kg diet), SPSeNPs (SeNPs + SP), and SPZnNPs (ZnNPs + SP). Means within a column not sharing a common superscript differ (*p* < 0.05); SEM standard error of means

### Effects on Blood Hematology

As shown in Table 5, the treatments had no significant effects on all erythrogram variables (except for Hb, RBCs, and Hct) and leucogram (except for WBCs). However, all treatments substantially (*p* < 0.05) boosted the serum values of WBCs, Hb, Hct, and RBCs compared to the CON group.

**Table 5:** Effects of nanominerals (SeNPs or ZnNPs) either alone or incorporated with *Spirulina platensis* (SP) on erythrogram and white blood cells of growing rabbits under summer conditions

Item^2^	Treatments^1^						SEM	*p*-value
	CON	SP	SeNPs	SPSeNPs	ZnNPs	SPZnNPs		
Hb (g/dL)	8.94^**b**^	11.22^**a**^	11.82^**a**^	12.31^**a**^	11.74^**a**^	11.38^**a**^	1.95	0.011
RBCs (10^6^/mm3)	4.77^**b**^	5.46^**a**^	5.25^**a**^	5.38^**a**^	5.36^**a**^	5.51^**a**^	0.11	0.036
Hct (%)	28.66^**b**^	32.74^**a**^	33.72^**a**^	42.26^**a**^	33.18^**a**^	32.58^**a**^	5.20	0.005
MCV (fL)	63.57	65.07	64.38	54.40	61.93	64.31	3.46	0.286
MCH (g/dL)	22.23	22.62	22.56	24.29	21.93	22.45	1.14	0.756
MCHC (%)	34.97	34.78	35.06	30.44	35.44	34.94	1.80	0.392
Platelets (10^3^/mm^3^)	186.60	182.40	201.40	173.00	204.00	216.00	27.32	0.883
MPV (fL)	7.21	6.71	6.99	9.93	7.20	6.75	1.37	0.567
PDW (fL)	24.38	19.82	21.96	20.04	25.76	24.96	2.97	0.595
WBCs (10^3^/mm^3^)	6.44^**b**^	9.98^**a,b**^	10.74^**a,b**^	8.83^**a,b**^	14.90^**a**^	9.62^**a,b**^	1.88	0.043

^1^Rabbits were given basal diet (CON group) or supplemented with *Spirulina platensis* (SP; 1g/kg diet), selenium nanoparticles (SeNPs, 50mg/kg diet), zinc nanoparticles (ZnNPs; 100 mg/kg diet), SPSeNPs (SeNPs + SP), and SPZnNPs (ZnNPs + SP). Means within a column not sharing a common superscript differ (*p* < 0.05); *SEM* standard error of means. ^2^Hemoglobin (Hb), red blood cells (RBCs), hematocrit (Hct), mean cell volume (MCV), mean corpuscular hemoglobin (MCH), mean corpuscular hemoglobin concentration (MCHC), platelets, mean platelet volume (MPV), platelet distribution width (PDW), and white blood cells (WBCs)

### Effects on Blood Biochemistry

The highest TP values were noticed in the SPSeNP group (*p* < 0.001), while the other treated groups did not show any significant differences in TP compared to the control group (*p* > 0.05; Table 6). Both groups (SeNPs and SPSeNPs) had the best values for GLU (*p* = 0.0004) compared to the groups, followed by the SPZnNP group. All treated groups showed a significant decrease in glucose, and TG levels compared to the CON group, with the lowest values detected in the SPZnNP group (*p* < 0.05). All dietary supplementations to growing rabbit diets significantly decreased serum glucose (*p* < 0.001), AST (*p* = 0.0113), ALT (*p* = 0.0013), creatinine (*p* = 0.0009), and uric acid (*p* = 0.0035) levels compared to the control group. With the exception of the ZnNP groups, all treated groups had lower values of TB and IDB in a dose-dependent way compared to the CON group. The values of DB were the lowest in both the SeNP and SPSeNP groups.

**Table 6:** Effects of nanominerals (SeNPs or ZnNPs) either alone or incorporated with *Spirulina platensis* (SP) on blood biochemical of growing rabbits under summer conditions

Item^2^	Treatments^1^						SEM	*p*-value
	CON	SP	SeNPs	SPSeNPs	ZnNPs	SPZnNPs		
TP (g/dL)	5.74^b^	6.32^ab^	9.01^ab^	9.59^a^	6.79^ab^	7.79^ab^	0.12	<0.0001
ALB (g/dL)	3.79	4.31	4.60	4.88	4.32	4.23	0.27	0.2536
GLU (g/dL)	1.95^c^	2.01^c^	4.41^a^	4.72^a^	2.47^c^	3.56^b^	0.22	0.0004
A/G	1.95	2.33	1.05	1.03	1.75	1.20	0.33	0.1375
Glucose (mg/dL)	88.58^a^	81.79^b^	80.20^b^	70.94^c^	79.43^b^	79.63^b^	1.11	0.0003
TG (mg/dL)	88.80^a^	64.32^b^	63.39^b^	46.23^c^	61.93^b^	63.83^b^	1.84	<0.0001
Creatinine (mg/dL)	1.79^a^	1.42^b^	1.34^b^	1.26^b^	1.51^b^	1.55^b^	0.04	0.0009
Uric acid (mg/dL)	7.88^a^	5.65^b^	5.29^b^	4.56^b^	5.40^b^	4.62^b^	0.33	0.0035
ALT (U/mL)	92.50^a^	80.46^b^	44.85^c^	45.9^c^	48.57^c^	41.37^c^	4.38	0.0013
AST (U/mL)	36.43^a^	26.83^b^	21.03^b^	25.43^b^	24.95^b^	24.92^b^	1.80	0.0113
TB (mg/dL)	0.92^a^	0.46^d^	0.67^c^	0.48^d^	0.89^a^	0.91^b^	0.03	0.0051
DB (mg/dL)	0.17^b,c^	0.14^c,d^	0.13^d^	0.12^d^	0.21^a^	0.19^a,b^	0.01	0.0020
IDB (mg/dL)	0.75^a^	0.32^d^	0.54^c^	0.36^d^	0.68^a^	0.72^b^	0.04	0.0012

^1^Rabbits were given basal diet (CON group) or supplemented with *Spirulina platensis* (SP; 1g/kg diet), selenium nanoparticles (SeNPs, 50mg/kg diet), zinc nanoparticles (ZnNPs; 100 mg/kg diet), SPSeNPs (SeNPs + SP), and SPZnNPs (ZnNPs + SP). Means within a column not sharing a common superscript differ (*p* < 0.05); SEM standard error of means. ^2^Total protein (TP), albumin (ALB), globulin (GLU), glucose, total triacylglycerol (TG), alanine aminotransferase (ALT), aspartate aminotransferase (AST), total bilirubin (TB), direct (DB), and indirect bilirubin (IDB)

### Effects on Antioxidant-Immune Responses

The values of IgA (*p* < 0.05) and IgG (*p* < 0.05) were significantly increased in the serum of treated rabbits compared with the CON group (Table 7).

**Table 7:** Effects of nanominerals (SeNPs or ZnNPs) alone or incorporated with *Spirulina platensis* (SP) on blood biochemical of growing rabbits under summer conditions

Item^2^	Treatments^1^						SEM	*p*-value
	CON	SP	SeNPs	SPSeNPs	ZnNPs	SPZnNPs		
IgA (mg/dL)	219.21^**c**^	277.50^**b**^	344.37^**a**^	361.70^**a**^	283.77^**b**^	373.41^**a**^	12.18	0.0414
IgG (mg/dL)	33.13^**c**^	38.17^**b**^	50.52^**a**^	54.63^**a**^	37.27^**b**^	49.62^**a**^	2.96	0.0396
T3 (ng/mL)	1.57^**b**^	2.88^**a**^	2.72^**a**^	2.69^**a**^	2.07^**a,b**^	2.53^**a**^	0.24	0.0121
Cortisol (ng/dL)	13.51^**a**^	10.22^**b**^	9.73^**b**^	10.61^**b**^	1112^**b**^	11.17^**b**^	1.22	0.0185`
IFN-γ (pg/mL)	67.00^**a**^	49.32^**b**^	52.50^**b**^	50.50^**b**^	52.50^**b**^	48.00^**b**^	3.21	0.0054
SOD (ng/mL)	1.64^**c**^	2.77^**a**^	2.24^**b**^	2.90^**a**^	1.81^**c**^	2.01^**b,c**^	0.11	0.0281
GSH (ng/mL)	0.17	0.29	0.37	0.39	0.25	0.33	0.10	0.3660
MDA (nmol/mL)	3.98^**a**^	2.98^**b**^	3.06^**b**^	1.62^**c**^	2.48^**b**^	1.60^**c**^	0.21	0.0014
PC (nmol/mL)	3.37^**a**^	2.32^**b**^	1.29^**b**^	1.87^**b**^	1.30^**b**^	1.72^**b**^	0.43	0.0295

^1^Rabbits were given basal diet (CON group) or supplemented with *Spirulina platensis* (SP; 1g/kg diet), selenium nanoparticles (SeNPs, 50mg/kg diet), zinc nanoparticles (ZnNPs; 100 mg/kg diet), SPSeNPs (SeNPs + SP), and SPZnNPs (ZnNPs + SP). Means within a column not sharing a common superscript differ (*p* < 0.05); SEM standard error of means. ^2^Triiodothyronine (T3), immunoglobulins (IgG and Ig G), interferon-gamma (IFN-γ), superoxide dismutase (SOD), glutathione (GSH), malondialdehyde (MDA), and protein carbonyl (PC)

The ZnNP, SPZnNP, and SPSeNP groups had greater levels of IgG and IgA compared with the other groups. Compared to the control, the serum levels of T3 (*p* < 0.05) were significantly increased in all treated growing rabbits (except the ZnNP group), while the serum cortisol (*p* < 0.05), IFN-γ, and PC levels were significantly decreased in all treated groups (*p* < 0.05). All supplemented groups had greater levels of SOD (*p* = 0.0281) and lower levels of MDA (*p* = 0.0014) in a dose-dependent way when compared to the CON group.

### Effects on Hepatic, Renal, and Cardiac Tissue Architecture

**Fig. 3:**
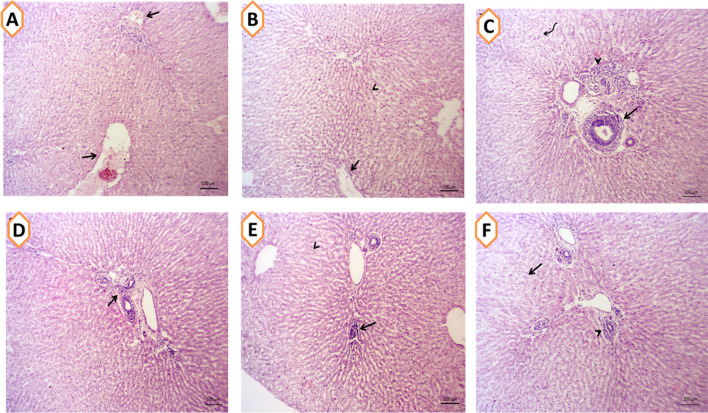
**A–F** The hepatic tissues of rabbit given basal diet (CON group; **A**) or supplemented with *Spirulina platensis* (SP; 1g/kg diet; **B**), selenium nanoparticles (SeNPs, 50mg/kg diet; **C**), zinc nanoparticles (ZnNPs; 100 mg/kg diet; **E**), SPSeNPs (SeNPs + SP; **D**), and SPZnNPs (ZnNPs + SP; **F**) reared under summer conditions

**Fig. 4:**
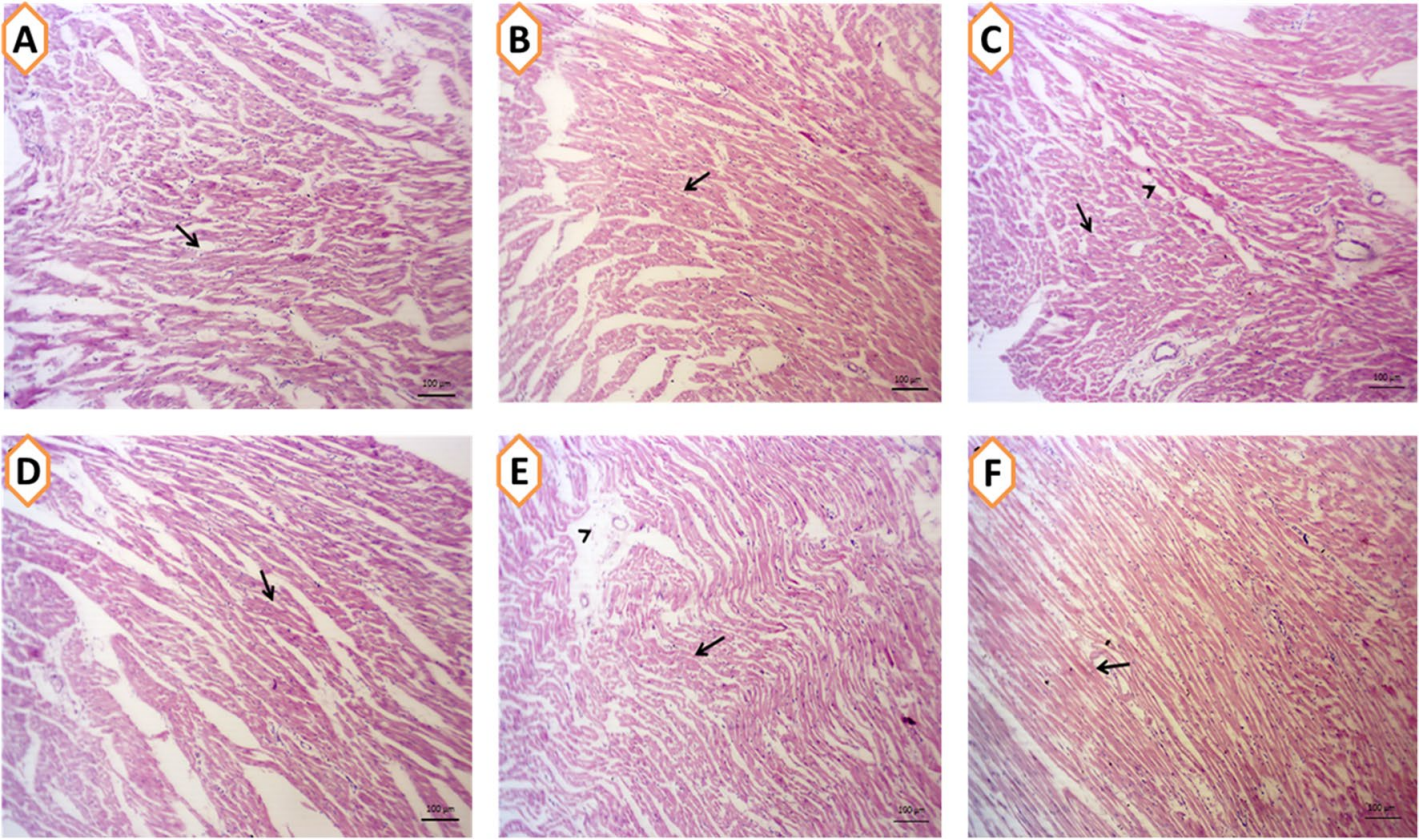
**A–F** The cardiac tissues of rabbit given basal diet (CON group; **A**) or supplemented with *Spirulina platensis* (SP; 1g/kg diet; **B**), selenium nanoparticles (SeNPs, 50mg/kg diet; **C**), zinc nanoparticles (ZnNPs; 100 mg/kg diet; **E**), SPSeNPs (SeNPs + SP; **D**), and SPZnNPs (ZnNPs + SP; **F**) reared under summer conditions

**Fig. 5:**
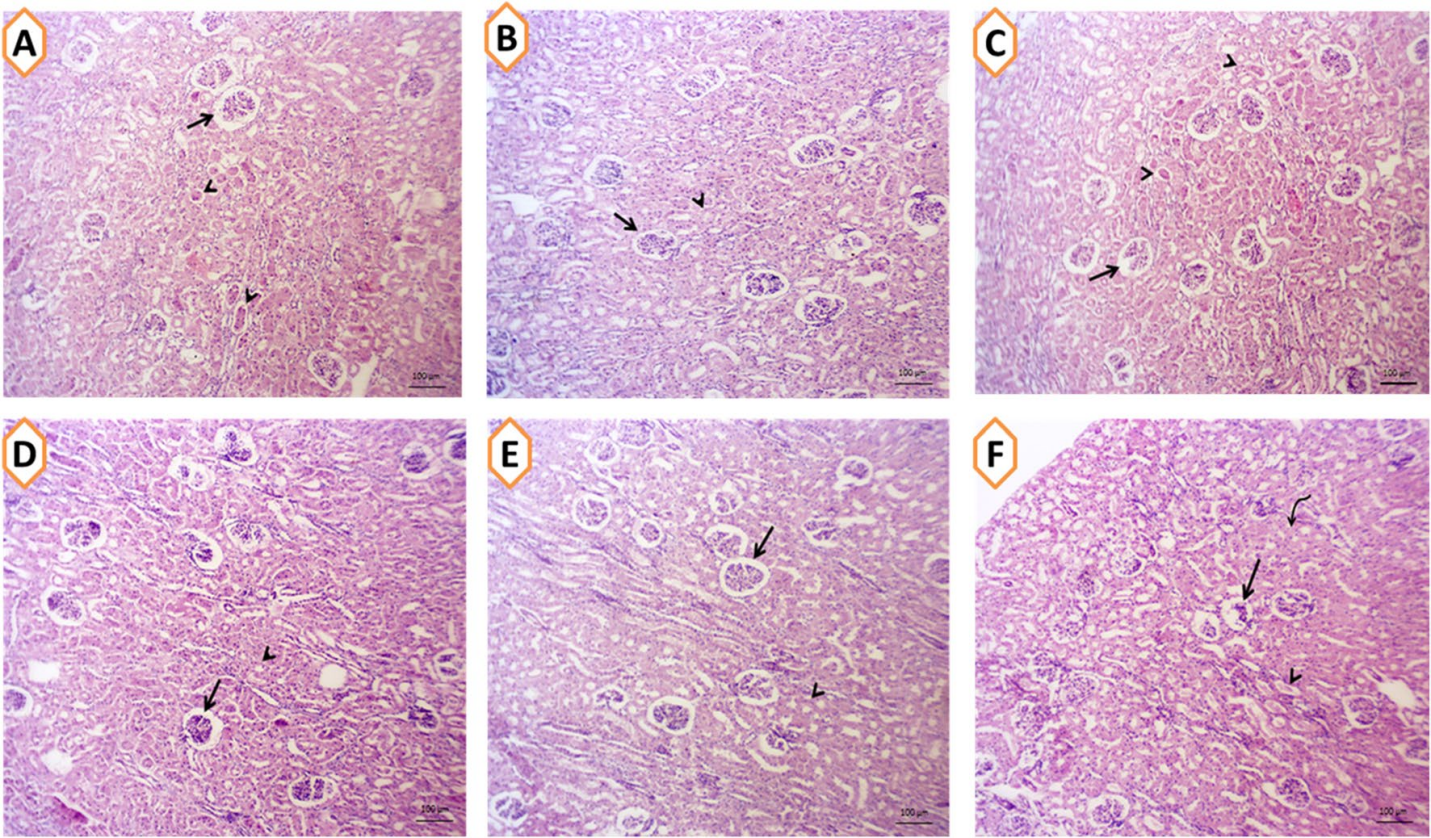
**A–F** The cardiac tissues of rabbits given basal diet (CON group; **A**) or supplemented with *Spirulina platensis* (SP; 1g/kg diet; **B**), selenium nanoparticles (SeNPs, 50mg/kg diet; **C**), zinc nanoparticles (ZnNPs; 100 mg/kg diet; **E**), SPSeNPs (SeNPs + SP; **D**), and SPZnNPs (ZnNPs + SP; **F**) reared under summer conditions

The impacts of nanominerals (SeNPs or ZnNPs) alone or incorporated with *Spirulina platensis* (SP) on hepatic (Fig. 3A–F), cardiac (Fig. 4A–F), and renal (Fig. 5A–F) tissues architecture of growing rabbits under summer conditions are shown in Figs. 3, 4, and 5. In Fig. 3A, rabbits (CON group) exhibited mild congestion of hepatic blood vessels with minute perivascular lymphocytic infiltrations, while rabbits treated with various feed additives showed normal hepatic cords, central veins, and hepatic sinusoids in hepatic tissues (Fig. 3B–F). As shown in Fig. 4A (CON group), rabbits exhibited some myocardial degeneration in the cardiac muscle fibers and cardiomyocytes with mild edema. Rabbits fed diets with nanomineral alone (Fig. 4C and E), mixed with SP (Fig. 4D, F) or alone (Fig. 4B), showed normal histomorphology of cardiomyocytes. Moreover, the cardiac tissue–supplemented groups showed apparently normal cardiomyocytes which appeared striated and branched with central round nucleus. The changes in renal architecture of stressed rabbits are shown in Fig. 5A–F). The rabbit exposed to HS had shrinkage of some glomeruli and necrotic few numbers of renal tubules as well as degenerative changes in few number of the cortical and medullary tubular epithelium (Fig. 5A). However, feeding rabbits with various nanominerals such as SeNPs (Fig. 5C), ZnNPs (Fig. 5E), SP alone (Fig. 5A), or mixed with SPSeNPs (Fig. 5D) and SPZnNPs (Fig. 5E) showed apparently normal nephron structures and renal parenchyma which represented by normal renal tubules and glomerular corpuscles.

## Discussion

Heat stress (HS) is a major environmental factor that has a negative impact on the health and immunity of rabbits. To sustain the productivity of growing rabbits in hot conditions, it is important to improve their health status and immune-antioxidant profiling. The current study suggests that including SP mixed with nanominerals (Zn or Se) in the diet can effectively enhance the growth indices and feed utilization of growing rabbits exposed to HS. This supplementation also supports immunity, improves health and serum biochemistry, and helps maintain the histoarchitecture of organs such as the heart, liver, and kidney in stressed growing rabbits.

When rabbits are exposed to high temperatures, their bodies work harder to maintain their internal temperature [39]. This can lead to a number of issues such as reduced feed intake and appetite, thus promoting weight loss and malnutrition.

Se and Zn are two essential minerals that are important for the growth and development of rabbits [29, 38]. They play a role in several processes, including protein synthesis, cell division, immune function, and antioxidant defense in the body’s cellular system [14]. Selenium is a powerful antioxidant that protects cells from damage caused by free radicals. It is also involved in the production of thyroid hormones, which are necessary for growth and development [18, 40]. As reported in previous studies similar with our data, the nanoform of selenium [12, 13, 15, 16] and ZnNPs [11, 12] exhibited significant improvement in the growth performance of stressed rabbits and broilers. Moreover, Se has been used in combination with SP in various studies to combat the negative effects of HS. To assess the health status of stressed rabbits after feeding nanominerals alone or mixed with SP, the blood hematology was evaluated. All treatments significantly improved the blood hematology variables. SeNPs (60 μg/kg) significantly improved the blood hematology in broilers during hot conditions [41]. Moreover, it has been reported that SP or its active compound (phycocyanin) significantly improved erythrocyte variables in stressed rabbits or exposed to lead sub-toxicity [10, 26]. These results are matching with the data of [20], who described that birds supplemented with ZnNPs in their diets had augmented WG and improved FCR than the control group. Also, [42] found that final BW, BWG, and FCR were enhanced by supplementation of ZnNPs (30–60 mg/kg) compared with the control group. However, in our data, we found the groups SeNPs, SP, or SPSeNPs exhibited more positive effects in the growth than the ZnNPs in rabbits subjected to HS. In contrast, [12] informed that adding zinc oxide (100 mg/kg) improved the performance of growing rabbits under HS conditions.

The results of this research may suggest that SeNPs or SP can improve rabbit growth and feed efficiency in multiple ways: they can enhance nutrient assimilation leading to improved growth. Moreover, these molecules can improve the secretion of digestive enzymes, helping the rabbits digest and absorb their food more efficiently. However, after reviewing the literature, we found no articles that compared these molecules in stressed growing rabbits.

Zinc has vital structural or catalytic functions in many metal-binding proteins and metalloenzymes, which are vital for growth [43], nutrient metabolism, immune system function [44, 45], and overall health [11] in rabbits. Additionally, Zn can enhance rabbit productivity by improving the expression of peptide growth factors and cytokine genes [43].

The carcass trait significantly improved after broiler feeding with SeNPs (100 mg/kg) and SP (10 g/kg diet) [15]. However, in our study, there was no significant effect on carcass traits. This difference might be associated with other authors who used high doses of SeNPs and SP, while in our study we used 50 mg of SeNPs and 1 g of SP. Studies have shown that zinc supplementation can improve hot carcass weight, dressing percentage, and total edible parts in rabbits [11, 15]. Additionally, supplementation of SeNPs (50 or 100 mg/kg) has been performed to improve carcass traits, such as hot carcass weight, dressing percentage, and meat quality in rabbits and broilers [12, 41]. This improvement may be due to the structural or catalytic roles of Zn in many metal-binding proteins and metalloenzymes. Furthermore, Zn has been proven to enhance the growth of rabbits by promoting the expression of peptide growth influences and cytokine genes [43]. In terms of the ability of Zn to improve growth in stressed rabbits, [46] found that Zn can maintain intestinal barrier function, thereby improving nutrient absorption to support growth. The principal mechanism to defend biological macromolecules from oxidative stress is the cellular enzyme defense system, which includes SOD, CAT, and GPx.

SeNPs can alleviate the negative impacts of HS by boosting antioxidant activity and reducing heat shock responses in various animals [12, 13, 15, 16]. Antioxidants help to defend cells from damage caused by OS-induced HS [28]. Free radicals are unstable molecules that can harm cells and tissues. During HS, the production of free radicals increases, so it is important for rabbits to have efficient levels of antioxidants. According to the data presented, the groups of SeNPs alone or incorporated with SP showed greater effectiveness in enhancing antioxidant biomarkers and reducing oxidative stress compared to the ZnNP and SPZnNP groups. In this regard, SP exhibits antioxidant activity by improving SOD, TAC, and GSH levels in the serum of rabbits fed diets containing SP [47, 48]. It was clarified that Zn may act as an antioxidant due to its role in the antioxidant defense system, regulating GPx and serving as a co-factor for SOD [46].

According to our data [28], it has been revealed that SeNPs or SPSeNPs can improve the heat tolerance of rabbit bucks [28] and broilers [15, 16] via supporting antioxidant defense and boosting immunity. In stressed broilers, [15] found that IgG and IgM levels were higher in birds fed supplemented diets compared to the HS group. These results confirm the immunomodulatory effect of Se [49], as it decreases the immune-inflammatory factors (IL-1β, TNF-α, and IL-6) in the rabbit kidney. Another explanation suggests that the Se can decrease oxidative stress, thus preserving cellular function [50]. ZnNPs can also help rabbits dissipate heat from their bodies, preventing heat stroke. Moreover, some studies have shown that ZnNPs (100 mg/kg diet) can improve the immune system in growing rabbits exposed to HS [22]. However, in contrast to the previous data, we showed little improvement in immunity after feeding with ZnNPs (100 mg/kg diet). In the current study, supplementation of SeNPs, SP, or SPSeNPs in the diets of stressed rabbits significantly decreased TG, ALT, creatinine, uric acid, AST, TB, DB, and IDB compared to the control and other treatments. Reducing these parameters to within normal values could indicate the health status of hepatic and renal functions [29, 38]. These enhancements in the serum biochemistry of growing rabbits may be attributed to the fact that Se and Zn are the main components of important enzymes, which are responsible for maintaining the structural integrity of liver and kidney cells [14, 51].

The immune system plays a crucial role in fighting off infections, which are more likely to occur during periods of heat stress. ZnNPs can help to boost the immune system, aiding rabbits in staying healthy during hot weather [22]. Studies have shown that ZnNPs can effectively mitigate the negative effects of heat stress on rabbits [22]. In one study, rabbits supplemented with ZnNPs exhibited a lower rectal temperature, reduced heart rate, and increased antioxidant capacity compared to rabbits that were not supplemented with ZnNPs [24]. The groups of rabbits supplemented with ZnNPs had a lower incidence of heat stress-related health problems. The dietary SP or SeNP treatments led to a significant increase in GSH and SOD levels, while resulting in a significant decrease in MDA levels in birds, as reported by [15].

HS can disrupt the normal functioning of the rabbit’s internal organs such as hepatic, cardiac, and renal tissues, leading to various physiological dysfunctions in the cellular system [52]. This condition is characterized by a slowdown or interruption of the digestive processes, potentially causing blockages in the liver and kidneys. In the current study, rabbits treated with feed supplements showed normal histomorphology of cardiomyocytes. Moreover, the cardiac tissue–supplemented groups showed apparently normal cardiomyocytes which appeared striated and branched with central round nucleus and regular nephron structures and renal parenchyma which was represented by normal renal tubules and glomerular corpuscles. Previous research has shown that certain phytochemicals can improve the hepatic structure of rabbits in hot climates [52] by supporting antioxidant defense mechanisms in the brain [39]. Another study of [53] found that SP or SeNPs alone or their mixture protected against hepatic injury caused by chronic alcohol. Lastly, the consequences strongly recommend enriched stressed growing rabbits diets with SP (1 g/kg diet) or SeNPs (50 mg/kg diet) alone or their mixture for promoting growth and health status under hot environments. To confirm these results, further research is needed, particularly using omics tools to investigate the effects of trace mineral nanoparticles incorporated into the diets of stressed rabbits.

## Conclusion

Overall, the results of this study validated that adding 1g of SP or SeNPS (100 mg of Se nanoparticles + 1 g of SP/kg diet) either individually or in combination to growing rabbits under summer conditions could improve growth performance, carcass criteria, antioxidant-immune status, and organs histoarchitecture. Therefore, supplementation of SP and SeNPs had beneficial effects on the health of stressed rabbits and may serve as a useful nutritional strategy against global warming. However, further investigations are needed to explore the molecular level and omics tools in response to various mineral nanoparticles in stressed rabbits.

## Data Availability

The original data in the article can be obtained directly from the corresponding author.
